# Exploring Ecological Alternatives for Crop Protection Using *Coriandrum sativum* Essential Oil

**DOI:** 10.3390/molecules24112040

**Published:** 2019-05-28

**Authors:** Renata Maria Sumalan, Ersilia Alexa, Iuliana Popescu, Monica Negrea, Isidora Radulov, Diana Obistioiu, Ileana Cocan

**Affiliations:** 1 Faculty of Horticulture and Forestry, Banat’s University of Agricultural Sciences and Veterinary Medicine “King Michael I of Romania” from Timisoara, Calea Aradului 119, 300645 Timisoara, Romania; srenata_maria@yahoo.com; 2 Faculty of Food Engineering, Banat’s University of Agricultural Sciences and Veterinary Medicine “King Michael I of Romania” from Timisoara, Calea Aradului 119, 300645 Timisoara, Romania; negrea_monica2000@yahoo.com (M.N.); negreaileana@yahoo.com (I.C.); 3 Faculty of Agriculture, Banat’s University of Agricultural Sciences and Veterinary Medicine “King Michael I of Romania” from Timisoara, Calea Aradului 119, 300645 Timisoara, Romania; iuliapopescu2002@yahoo.com (I.P.); isidoraradulov@yahoo.com (I.R.); 4 Interdisciplinary Research Platform, Banat’s University of Agricultural Sciences and Veterinary Medicine “King Michael I of Romania” from Timisoara, Calea Aradului 119, 300645 Timisoara, Romania; diana.obistioiu@yahoo.com

**Keywords:** antifungal, GC-MS, bioherbicide, deoxynivalenol, *Fusarium graminearum*, weed

## Abstract

Essential oils (EOs) are a natural source of active compounds with antifungal, antimycotoxigenic, and herbicidal potential, and have been successfully used in organic agriculture, instead of chemical compounds obtained by synthesis, due to their high bioactivity and the absence of toxicity. The aim of this study was to highlight the importance of *Coriandrum sativum* essential oil (CEO) as a potential source of bioactive constituents and its applications as an antifungal and bioherbicidal agent. The CEO was obtained by steam distillation of coriander seeds and GC-MS technique was used to determine the chemical composition. Furthermore, in vitro tests were used to determine the antifungal potential of CEO on *Fusarium graminearum* mycelia growth through poisoned food technique, resulting in the minimum fungistatic (MCFs) and fungicidal concentrations (MCFg). The antifungal and antimycotoxigenic effect of CEO was studied on artificially contaminated wheat seeds with *F. graminearum* spores. Additionally, the herbicidal potential of CEO was studied by fumigating monocotyledonous and dicotyledonous weed seeds, which are problematic in agricultural field crops in Romania. The in vitro studies showed the antifungal potential of CEO, with a minimum concentration for a fungistatic effect of 0.4% and the minimum fungicidal concentration of 0.6%, respectively. An increase in the antifungal effects was observed in the in vivo experiment with *F. graminearum*, where a mixture of CEO with *Satureja hortensis* essential oil (SEO) was used. This increase is attributed to the synergistic effect of both EOs. Moreover, the synthesis of deoxynivalenol (DON)-type mycotoxins was found to be less inhibited. Hence, CEO has shown an herbicidal potential on weed seeds by affecting inhibition of germination.

## 1. Introduction

Crop contamination with fungal species during the vegetation period and postharvest, under inadequate storage conditions, such as high air humidity and poor or absent ventilation, is a common issue and results in significant economic losses [1]. Replacement of synthetic compounds used as protective agents during crop vegetation or storage, with natural, non-toxic, and environmentally-friendly compounds is desirable in organic agriculture. In this regard, essential oils (EOs) of aromatic plants are being explored as alternative herbicides or fungicides in organic agriculture and horticulture. Their major advantage is that they do not contaminate groundwater or persist in soil and, more importantly, they do not cause mammalian toxicity [2]. Various arguments regarding the mechanisms of volatile oils activity on the microbial cell have been developed over time. However, the fact is that they can affect both the integrity and the functionality of the cytoplasmic membrane. Their hydrophobic (lipophilic) character appears to be responsible for disturbing microbial lipid structures by increasing the permeability of the cytoplasmic membrane, affecting the functionality of the cell [3].

Coriander (*Coriandrum sativum*) is an annual spice herb from the Apiaceae family, with all plant parts edible, possessing nutritional and medicinal properties. Several biological activities, which include antimicrobial, antioxidant, hypoglycemic, analgesic, anti-inflammatory, and anti-carcinogenic activities, have been reported in previous studies, alongside the chemical composition of the bioactive components [4,5]. The antifungal properties of coriander essential oil (CEO) have been reported on for pathogenic species such as *Candida albicans* [6,7] and also on molds like *Alternaria alternata*, *Stachybotrys chartarum*, *Cladosporium cladosporioides*, and *Aspergillus niger* [8]. Additionally, the raw extract of coriander seeds has been tested as an antifungal agent against *Penicillium lilacinum*, *Aspergillus niger* [9], and *Leucoagaricus gongylophorus* [10].

*Fusarium* sp. represents one of the most important phytopathogenic fungi, which infects grains and produces toxic secondary metabolites, resulting in harmful contamination of feedstuffs and foodstuffs. Furthermore, the two diseases caused by *Fusarium* spp.—head blight (FHB) of small cereals (wheat, barley, oat) and ear rot of maize (FER)—lead to toxicological contaminations and economic losses [11]. Contamination of cereals with *Fusarium* spp. is an important risk factor for health because of the high mycotoxin production potential derived from these contaminations, which by consumption leads to hepatic toxicity, immunosuppression, allergenic reaction, and teratogenic, mutagenic, and carcinogenic effects [11]. Until now, positive results have been reported regarding the antifungal effect of different EOs on the development of *F. oxysporum*, *F. culmorum* and *F. solani* [12,13,14,15,16]. Secondary metabolites are toxic to humans and animals, with the most common mycotoxin produced by *F. graminearum* being deoxynivalenol (DON). In our previous study, we have demonstrated the in vivo effect of EOs obtained from medicinal plants belonging to the Lamiaceae family against naturally occurring fungi on grain seeds [17].

Recently, the use of EOs has been proposed as an alternative resource to synthetic chemical compounds against microbial attack or for the inhibition of weed germination [18,19,20,21]. Terpenic compounds of EOs have proven to be as effective as natural herbicides due to the fact that they lead to an inhibition of weed growth. Hence, pyrethrins as well as cineol, derived from cinmethylamine, are considered in the development of new pesticides. The antimicrobial or herbicidal effects may lead to enhanced properties by combining different properties of medicinal plants. Therefore, the aim is to achieve natural bio-pesticides from plant mixtures with different biological activity. Our previous study has shown the enhanced antimicrobial and allelopathic properties of the *Salvia officinalis* EO in a mixture with *Thymus vulgaris* oil [20].

The antimicrobial, including the antifungal potential, of CEO has been studied and proven on *Candida* spp. [7]. The in vivo effect of CEO against *F. graminearum* was generally limited, despite the fact that these plant pathogens produce significant losses in wheat and barley crops (in some years the damage raises up to 65–80%) [11]. To the best of our knowledge, the potential of CEO to inhibit the germination of weed seeds has not been reported on yet.

The aim of this study is to highlight the potential use of CEO as an alternative to synthetic pesticides in organic technologies. In this regard, our study focused on the in vitro and in vivo antifungal potential of CEO against *F. graminearum*, as well the bio-herbicide potential against weed seed germination of *Amaranthus retroflexus* (ARET), *Chenopodium album* (CALB), and *Echinochloa crus-galli* (EGAL). In addition, in light of recent research on the synergy of EOs [19], an increase in CEO performance was sought by the addition of *Satureja hortensis* essential oil (SEO) in various ratios.

## 2. Results and Discussions

### 2.1. The Chemical Composition of CEO

The mean value of CEO yield was found to be low (0.6%), this being in accordance with previously reported studies on the amount of CEOs in seeds in the range from 0.2% to 1.5% [22]. The GC-MS profiles of CEO chemical composition and concentration (in order of elution) are listed in Table 1. In CEO, twenty-one compounds were identified comprising 99.76% of the total EO composition. Monoterpene hydrocarbons (MH) were identified at 39.204%, while monoterpene oxygenates (MO) represented 60.556% of the total compounds. The chromatographic profile of CEO showed that linalool was the major compound, at 45.387%, followed by α-pinene, at 11.626%, and D-limonene, at 9.628% (Table 1). Other compounds identified with a concentration higher than 5% were p-cymene (8.00%), camphor (6.016%), and γ-terpinene (5.236%).

Our results are consistent with previous investigations regarding the chemical composition of CEO that reported the linalool as the major constituent of CEO. However, the values vary greatly depending on geographic area, climate, and cultivation practice. For example, in CEO obtained from *C. sativum* seeds cultivated in Cuba, the content of linalool reached 54.56%, while the seeds from Brazil contained 77.48% linalool [22]. The group of Zamindar et al., 2016, found linalool (73.05%) as the main chemical compound in CEO, followed by α-pinene (9.18%), γ-terpinene (7.65%), and geranyl acetate (2.71%) [23]. In a study published by Momin et al., 2012, the major component of CEO was linalool (60–70%), followed by α-pinene, limonene, γ-terpinene, *p*-cymene, borneol, citronellol, camphor, geraniol, and geraniol acetate [24]. Similar monoterpene composition (linalool 58.22%, geraniol 17.87%, and geranyl acetate 12,2%) was determined in Brazilian CEO [25]. CEO analyzed in the study of Al-Snafi et al., 2016, obtained from seeds of *Coriandrum sativum* was also defined by a relatively high amount of linalool (55.09%) followed by α-pinene (7.49%) and geraniol (4.83%) [26]. In contrast, the chromatographic profile of the CEO obtained from Romanian seeds contained 45.387% linalool. From this point of view, we can say that the coriander oil produced in Romania has less linalool content in comparison to the rest. A different chemical composition was found in CEO extracted from seeds cultivated in Iran, where camphor was the main chemical compound (44.99%), followed by cis-2-tert.butyl-cyclohexanol acetate (14.45%), limonene (7.17%), and α-pinene (6.37%) [27].

Previous studies have shown that the chemical composition of CEO varies depending on which part, vegetative or generative, is used in the steam distillation [28]. De Freires et al., 2014, found that CEO obtained by steam distillation of *C. sativum* leaves contained decanal (19.09%), *trans*-2-decenal (17.54%), 2-decen-1-ol (12.33%,) and cyclodecane (12.15%) [7]. The CEO extracted from leaves has a lower content of linalool compared to oil extracted from seeds. Yildiz et al., 2016, reported in a study conducted in Turkey that CEO obtained from leaves contains 21.61% linalool, while other compounds were (*E*)-2-decenal (29.87%), (*E*)-2-dodecenal (7.03%), dodecanal (5.78%), (*E*)-2-undecenal (3.84%), (*E*)-2-tridecenal (3.56%), (*E*)-2-hexadecenal (2.47%), tetradecenal (2.35%), and α-pinene (1.64%) [29].

### 2.2. The Antifungal Activity Effectiveness

**Figure 1:**
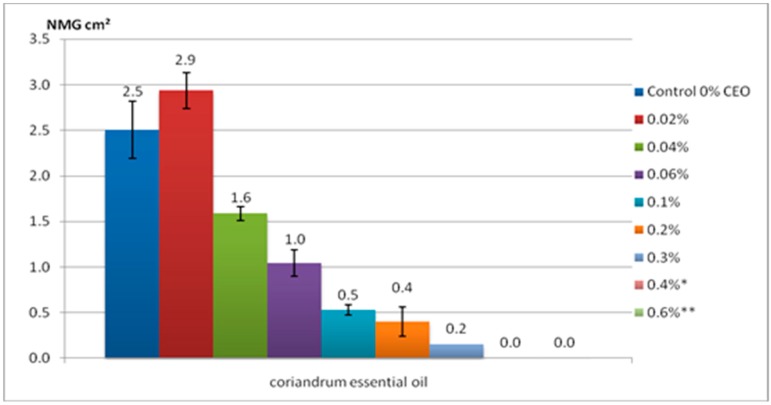
The new mycelium growth areas (NMG) on the medium at different concentrations of coriander essential oil (CEO); * Minimal concentration with fungistatic effect (MCFs value); ** Minimal concentration with fungicide effect (MCFg value). Data are mean ± SD, *n* = 6.

The CEO added in the culture medium inhibited the growth of new *F. graminearum* mycelium starting at a concentration of 0.04%. As can be seen in Figure 1, the new mycelial growth (NMG) values vary between 2.9 cm^2^, corresponding to the experimental sample treated with 0.02% CEO, and 0.2 cm^2^ for 0.3% CEO. On the other hand, in the case of a sample with 0.2% CEO, the NMG value exceeded the NMG control value by 0.4 cm^2^. It seems that the presence of reduced amounts of CEO stimulates the *F. graminearum* mycelia growth. Only from 0.04% CEO did we observe a decrease in the mycelinum area. The decrease was observed progressively with increasing CEO concentration. Thus, at 0.3% CEO, the NMG value reached only 0.2 cm^2^. As shown above, 0.4% CEO corresponded to minimal fungistatic effect (MCFs). The minimum concentration for fungicidal effect (MCFg) corresponded to 0.6% CEO. The MCFg value was confirmed only after transfers of mycelial disks and that the fungus hyphae had not been revived. This concentration was used for in vivo antifungal testing of the wheat kernel by fumigation with CEO. The antifungal effect of the CEO could be attributed to the linalool compound, which was reported to have an antibacterial effect [31], and is responsible for the flavor of the coriander [22]. The previous studies showed that CEO has an excellent antifungal activity against seed borne pathogens of paddy: *Pyricularia oryzae, Bipolaris oryzae, Alternaria alternata, Tricoconis padwickii, Drechslera tetramera*, *D. halodes, Curvularia lunata, Fusarium moniliforme*, and *F. oxysporum*. The fungicidal effect of CEO against *F. oxysporum* was 100% at a level of 2000 µg/mL and 25.55% at 500 µg/mL [22]. Zare-shehneh et al. (2014) showed that the MCFs against *P. lilacinum* and *A. niger* were 67.8 and 62.1 mg/mL, respectively [32].

Consequently, variable amounts of linalool in the CEO, suggests the necessity of the addition of a further plant oil extract [33]. In this way, a variety of active components would ensure the inhibition of pathogen or decay microorganisms, which cannot develop a resistance, and antimicrobial or antifungal efficiency.

### 2.3. Effect of Augmented CEO against the Growth of Mycotoxigenic Fungi and Deoxynivalenol (DON) Accumulation

**Figure 2:**
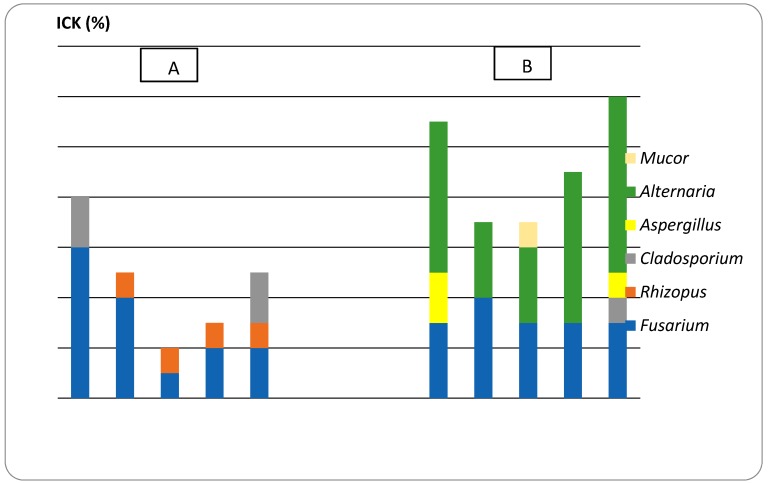
The index of colonization of wheat kernels (ICK, %) after (**A**) 7 days and (**B**) 14 days of fumigation with EOs; CEO (0.6%), CS1 (CEO 10% + SEO 2%), CS2 (CEO 1% + SEO 0.2%), CS3 (0.5% + SEO 0.1%). Data are presented as mean ± SD from two independent experiments (*n* = 2).

Recently, the synergistic effects of EO mixtures, with other EOs [34] or standard antimicrobial agents [35] have been reported. Both studies have proven that mixtures enhance the desired properties significantly. The seed contamination index (ICK, %) for all the fungal species identified in the case of wheat kernels fumigated with CEO and CEO/SEO mixture after 7 and 14 days, respectively, from treatment are presented in Figure 2.

After 7 days of fumigation, a significant decrease in the number of wheat kernels contaminated with *Fusarium graminearum* was observed when compared to the control experiment. The lowest value recorded in the CS1 mixture was an ICK value of 10%. This suggests a synergistic antifungal effect of CEO with SEO in controlling *Fusarium* contamination in the studied wheat kernels. *Rhizopus* sp., along with *F. graminearum*, are tolerant to EO vapors. Hence, the presence of fungal contamination at 7 days after fumigation in all samples studied, regardless of the ratios, was observed.

After 14 days of fumigation, a decrease from 60% to 40% of the ICK value for *Fusarium* in the presence of the CEO was observed. At the same time, the appearance of the *Alternaria* sp., denotes the exhaustion of the antifungal effect of EOs, probably due to loss of antifungal compounds by volatilization.

Other studies proved that the CEO has an antifungal effect at various concentrations over *Alternaria alternata, Stachybotrys chartarum, Cladosporium cladosporioides*, and *Aspergillus niger* [8]. The antifungal activity effect of coriander extracts was also proven by Zardini et al. (2012) by studying its effects on *P. lilacinum* and *A. niger* [9].

The use of EOs for grain storage by fumigation is a popular technique nowadays. Thus, *Syzygium aromaticum* and *Vatica diospyroides* EOs have proven to be very effective against *Aspergillus flavus* on maize seeds fumigated with 10 µl/L, even if the dose was found to subsequently affect germination capacity of the seeds [36].

In our research, contamination with *Fusarium* did not decrease after 14 days, highlighting that the treatment effect of CEO in fungal control, even by mixing with SEO, is maintained for only 7 days, after which a second treatment is needed. Additionally, the samples fumigated with CEO alone or combined with SEO in the CS1 and CS2 mixtures, and also in the control sample, the appearance of *Aspergillus, Mucor*, and *Cladosporium* can be noticed. Promising results regarding the use of CEOs in mixtures has proven antifungal protection by mixing 800 ppm CEO with 250 ppm *Cinnamomum cassia*, from the Lauraceae family against *Byssochlamys fulva* [23]. In the case of the CS3 mixture, a minimal fungus load with *Fusarium* sp. and *Alternaria* sp. correlated with economic efficiency, which recommends this conceptual solution for the fungal protection of cereals in storage, is noted.

SEO, used as additional compounds in our study, contains, as major compounds, o-cymene (30.728%), thymol (25.746%), and γ-terpinene (11.821%). Other compounds were identified in concentrations lower than 10%. The group of Katar et al. (2017) reported γ-terpinene and carvacrol as major components and highlighted that the climatic and geographic conditions influence the chemical composition of SEO [37].

**Figure 3:**
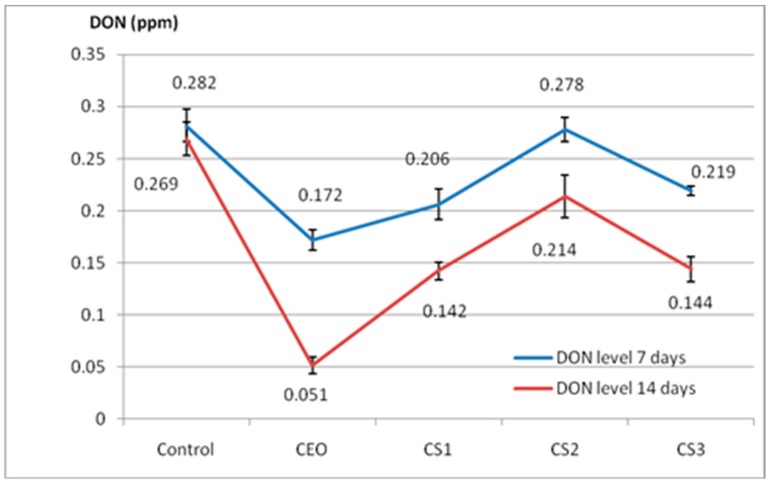
Deoxynivaleno (DON) levels (ppm) in wheat seeds determined after 7 and 14 days of fumigation with essential oil. CEO (0.6%), CS1 (CEO 10% + SEO 2%), CS2 (CEO 1% + SEO 0.2%), CS3 (0.5% + SEO 0.1%). Data are mean ± SD, *n* = 3.

The synergistic or antagonistic effects generated by the presence of chemical compounds in the two EO compositions may be responsible for the different biological action of CEO/SEO mixtures when compared to the pure CEO. The inhibitory effect of CEO oil is due to oxygenated monoterpenes. The experimental results are shown in Figure 3.

The DON content in the control sample after 14 days was 0.269 ppm, with the decrease in the mycotoxigenic load being 4.6%. This decrease is in line with the evolution of *F. graminearum*, whose development decreases over time with the occurrence of other fungal species, such as *Alternaria* sp. and *Mucor* sp. in wheat samples. The treatment with CEO, led to a considerably decreased DON content, compared with the control (39%) after 7 days. This decrease was even more pronounced after 14 days (81.04%). The treatment with CS leads to a similar profile to CEO treatment, however, the inhibition effectiveness of DON formation is lower compared to CEO treatment. In the case of treatment with CS mixture, the reduction in DON contamination of samples when compared to the control varied between 1.41% and 26.95%. Furthermore, the CS mixture showed the optimal protection against the synthesis of DON mycotoxin.

It was observed that there is no linear dependence between the CS concentration and the DON content. The more concentrated mixtures (CS2) led to a higher mycotoxigenic load (0.278 ppm) compared to the more diluted (0.216 ppm) CS3 sample. The rate of growth inhibition after 14 days of CEO treatment and mixtures varied between 81.04%, in the case of CEO treatment, and 29.44% for the cereals treated with the CS2 mixture. It is important to emphasize that the SEO addition to the CEO did not induce the mycotoxins synthesis, as the inhibition effects of DON synthesis were lower for CS variants. Similar results on the inhibition of DON production with EO treatment belonging to Lamiaceae family have been previously reported, which showed that, after 22 days of treatment with EOs, DON was undetectable in wheat samples [17,38].

### 2.4. The Herbicidal Potential Assay

**Figure 4:**
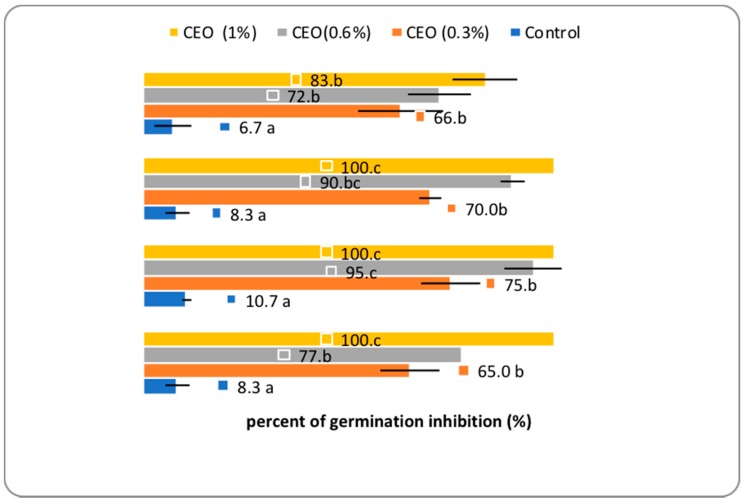
The percent of seed germination inhibition in the presence of CEO (1%, 0.6%, and 0.3%). Between the mean values followed by different letters, there are statistical differences assured for *p* < 0.05 by *t*-Test (*n* = 3) WHEAT, (*Triticum aestivum*), EGAL (*Echinochloa crus-galli*), CALB (*Chenopodium album*), and ARET (*Amaranthus retroflexus*).

The herbicidal potential of CEO has been tested for weed seeds using the fumigation technique to inhibit germination. The herbicidal potential has been observed on monocotyledonous weed seeds, *Echinochloa crus–galli* (EGAL), and has been extended to wheat seeds (WHEAT). Among dicotyledonous weeds, this study focused on the problematic weeds known for their aggressiveness and competitiveness in the crop field, i.e., *Amaranthus retroflexus* (ARET) and *Chenopodium album* (CALB). To the best of our knowledge, this work presents the first bio-herbicidal study of CEO from Romania and the results are displayed in Figure 4. The rate of inhibition of weed germination in the case of fumigation with CEOs in different concentrations (0.3%, 0.6%, and 1%) varied between 65% and 100%. At 1% CEO concentration, the herbicidal effect was maximal for all weed seeds. *Chenopodium album* showed the highest sensitivity among the tested weeds, with 75% and 95% inhibition rate for the CEO at 0.3% and 0.6%, respectively. The effect of CEO on wheat seed germination presents various germination inhibition behavior with values between 66% and 83%. The maximum inhibition was recorded at 1% CEO.

**Figure 5:**
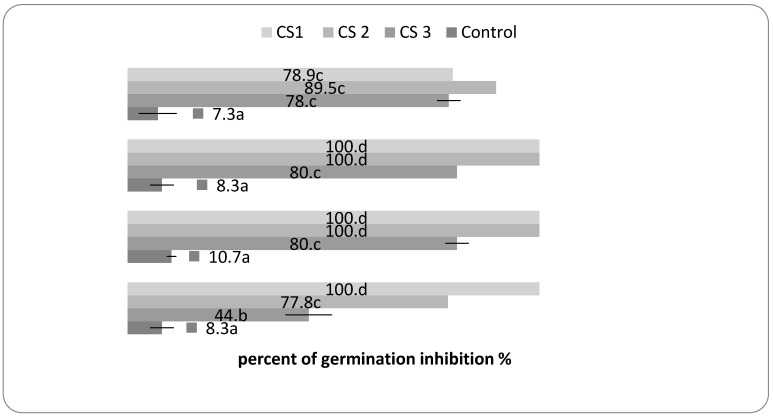
The percent of seed germination inhibition in the presence of CS1 (CEO 10% + SEO 2%), CS2 (CEO 1% + SEO 0.2%), and CS3 (0.5% + SEO 0.1%). Between the mean values followed by different letters, there are statistical differences assured for *p* < 0.05 by *t*-Test, *n* = 3. WHEAT, (*Triticum aestivum*, Antille cultivar), EGAL (*Echinochloa crus-galli*), CALB (*Chenopodium album*), ARET (*Amaranthus retroflexus*).

The CS mixture revealed a 100% germination inhibition effect for all three seed types analyzed when used in ratios of 10% CEO + 2% SEO. The CS2 mixture presented a 100% inhibition of germination of CALB and EGAL, and 77.8% of ARET. The lower concentration, CS3, inhibited the seed germination with values between 44% and 80%. In the case of wheat seeds, germination was inhibited 78.9–89.5%, depending on the CS concentration, see Figure 5. The variation of the herbicidal effect of EOs on seeds germination may be due to the different seed coat layers and their permeability [39].

Our study confirms previous results with regards to the herbicidal effect of EOs, which would recommend them as natural herbicides in organic farming. Regarding the allelopathic effect of EOs in connection with wheat germination, previous studies have shown that wheat cultivars were less affected compared to weed species, suggesting the possibility to use these natural compounds—in proper doses—as bioherbicides for weed control [40,41]. However, the germination inhibition rate of rosemary EO for wheat cultivars ranged between 10.3% and 78.5%, when the concentration dose was in the range of 2 to 16 μL.

Until now, there has been no studies reporting on the herbicidal effect of the CEO. The allelopathic effect of EOs extracted from different medicinal and aromatic plants was reported by Paudel and Gupta (2009) against seed germination of *Parthenium hysterophorus* L., a noxious weed [42]. The results have shown that lemongrass at 8 mL/L, and cinnamomum and eucalyptus oil at 12 mL/L, inhibited the germination of *Parthenium* seeds completely. Other studies reported that cinnamon EO exhibited the stronger inhibition effect on the seed germination of redroot pigweed and wild mustard, while peppermint oil has been the most effective in inhibiting ryegrass seed germination [43].

Oregano and rosemary EOs exhibited efficient control against the development of *Avena sterilis* and *Sinapis arvensis* weeds, commonly found in the wheat growing field. Even an application of 2 μL EO had a harmful effect. The inhibition rate of *S. arvensis* was 100% when 4 μL of oregano EO was applied, while the inhibition rate of *A. sterilis* was 64.6% with the application of a 16 μL dose of rosemary oil. Furthermore, the inhibition rates increased with increased oil concertation [40].

## 3. Materials and Methods

### 3.1. Extraction and GC-MS Characterization of CEO

*Coriandrum sativum* seeds were purchased from the Agricultural Research Development Resort Secuieni, Neamt County, Romania (46°51′45″ N 26°49′42″ E). The seeds of *Coriandrum sativum* were ground with Grindomix Retsch GM 2000 laboratory mill (Haan, Germany) and 300 g of a homogenous sample were used for steam distillation for 2.0 h, using Clevenger-type equipment (Timisoara, Romania), according to the European Pharmacopoeia (1975) [44]. The obtained oil was stored at 2–4 °C until the antifungal and herbicidal analysis.

The extraction yield of the CEO was calculated using the following formula:

% yield = [amount of CEO (g)/amount of seeds (g)] × 100.(1)

The chemical characterization of CEO was done using the gas-chromatograph equipment with mass spectrometer (GC-MS) Shimadzu QP 2010Plus with a capillary column with the characteristics: AT WAX 30 m × 0.32 mm × 1 μm. The carrier gas used was helium with a flow rate of 1 mL/min. The settings used for the separation was: 40 °C for 1 min, a rate of 5 °C/min to 210 °C for 5 min. Injector and ion source temperatures were 250 °C and 220 °C, respectively. The injection volume was 1 μL at a ratio of 1:50.

The identification of the chemical composition of CEO volatile compounds was performed in accordance with other specialized studies using 3 alternative methods: comparison with NIST 5 Wiley 275 libraries database, calculation of the linear retention index (LRI), and comparison with LRI from the literature data. The linear retention indices (LRIs) were determined in relation to a homologous series of n-alkanes (C8–C24) under the same operating conditions and calculated according to Van den Dool and Kratz formula and compared with the literature data [30,45].

### 3.2. In Vitro Antifungal Activity Assay

The in vitro study aimed to determine the minimum CEO concentration with impact on mycelia growth, through the “poisoned food technique” [46]. In order to study the fungal mycelium’s ability to grow and regenerate, a defined amount of CEO was added to the culture medium. The culture medium used was YCGA (yeast extract glucose chloramphenicol agar, 95765, Sigma-Aldrich), with the oil being added in different quantities to achieve the following concentrations: 0% (control), 0.02%, 0.04%, 0.06%, 0.1%, 0.2%, 0.3%, 0.4%, and 0.6% CEO (*v*/*v*). Minimum concentration with fungistatic effect (MCFs) is defined as the concentration starting from which the fungal growth is inhibited. By transferring the fungus to a fresh medium (without EO addition) a revival of hyphae and the mycelium growth is observed. Minimum concentration with fungicidal effect (MCFg) is defined as the concentration from which the fungal growth is inhibited and by transferring the inoculum to a fresh medium there is no hyphae or mycelium revival.

9 mm disks of 4-day-old *F. graminearum* mycelium were transferred to the YCGA medium with different concentrations of CEO (three of each concentration taken as repetitions). Incubation was done under alternate light/dark conditions 12 h/12 h. On the 5th day, observations were made by measuring two perpendicular diameters for each new fungal growth. The new mycelia growth (NMG) was calculated using the following formula:

NMG (cm^2^) = [(DM2 × 3.14/4) − IMD]/100 (2)

where DM is the diameter of finally mycelium growth (mm) and IMD is the initial mycelium disc (0.63 mm^2^). The experiment for antifungal activity assay was conducted twice.

### 3.3. In Vivo CEO Potential against Mycotoxigenic Fungi and DON Accumulation

Organic wheat kernels, Antille cultivar, harvested in 2016 were used to evaluate the effect of CEO on the growth of mycotoxin fungi and the synthesis of deoxynivalenol mycotoxin. Therefore, the fumigation technique of wheat grain in sealed flasks (Schnelldorf, Germany) was used. Firstly, the kernels were artificially inoculated with an *F. graminearum* spore suspension with 2 × 10^6^ CFU/mL certified with Bürker-Türk hemocytometer chamber BLAUBRAND (Wertheim, Germany). After 2 days, 50 g of wheat kernels samples were distributed in flasks lined with filter paper to which 5 mL of the studied oil treatment variant were added. The flasks were hermetically sealed and placed at 20–22 °C, in the dark.

To highlight CEO’s potential against mycotoxigenic fungi, DON accumulation and herbicidal effect mixtures with SEO in various volumetric rapports were used. SEO was obtained and evaluated in our laboratory by GC-MS, and the major compounds found were carvacrol (45.7%), p-cimene (12.6%), and γ-terpinene (8.1%). [47].

The different ratios used for the fumigation of kernel were: CEO (0.6%, *v*/*v*), CS1 (CEO 10% + SEO 2%), CS2 (CEO 1% + SEO 0.2%), and CS3 (0.5% + SEO 0.1%). CS1, CS2, and CS3 were achieved by mixing EOs in volume ratios (*v*/*v*). After 7 and 14 days, respectively, the colonization of fumigated wheat grains was evaluated by placing 10 wheat kernels from each ratio on CYGA medium in Petri dishes incubated on day/night regime at 22 + 2 °C. After 4 days, the index colonization value of wheat kernels (ICK) was determined for each treatment [48]:

ICK (%) = (NCK/NTK) × 100(3)

where NCK is the number of contaminated kernels with one fungus species/treatment and NTK is the number of total kernels used on treatment.

Assay of DON content was performed by the immunoenzymatic method (ELISA) after 7 and 14 days of seed fumigation in the presence of the studied EOs. Sample preparation was carried out according to the manufacturer’s instructions for DON analysis in cereals (R-Biopharm) using an ELISA 96 reader (PR-1100, Bio-Rad Laboratories, Swindon, UK). Each sample, including standards, was analyzed twice. The average content of DON (ppm) initially determined in the wheat samples was 0.464 ppm; the sample humidity was 12.4% for water activity of a_w_ = 0.9.

### 3.4. The Herbicidal Potential Assay

Weed seeds of *Amaranthus retroflexus* (ARET), *Chenopodium album* (CALB), and *Echinochloa crus-galli* (EGAL) were obtained from the seeds collection of the Department of Herbology, Faculty of Agriculture Timisoara. Seed samples were sterilized using a sodium hypochlorite solution (hypochlorite/water 1:9) for 3 min. Subsequently, the seeds were washed 3 times with distilled water for 3 min each time. A total of 10 seeds from each species were placed in a Petri dish, in layers separated by thin filter paper. Then, the seeds were treated with 6 mL of CEO (*v*/*v*) in the following concentrations: 0.3%, 0.6%, and 1%. To study the inhibition potential on germination of the weed seeds, the following mixtures of 3 mL CEO (*v*/*v*) + 3 mL SEO (*v*/*v*) were used: CS1 (CEO 10% + SEO 2%), CS2 (CEO 1% + SEO 0.2%), and CS3 (0.5% + SEO 0.1%). CS1, CS2 and CS3 were achieved by mixing EOs in volume ratios (*v*/*v*). Additionally, a control experiment was used, utilizing only distilled water (6 mL). To prevent the water loss, the Petri dishes were sealed with adhesive tape. The Petri dishes were kept at 25 °C in darkness and verified daily and on the 7th day the inhibition was evaluated. Each treatment, including the control, was repeated three times (in total 36 Petri dishes were used).

### 3.5. Statistical Analysis

The experimental assays were repeated twice (Section 3.1, Section 3.2, Section 3.3) and thrice (3.4). The results are presented as the mean ± standard deviation (SD). Statistical processing of data was performed using Microsoft Excel 2010. Significant statistical differences of investigated parameters were determined by *t*-Test: two-sample assuming unequal and equal variances at *p* < 0.05, after analysis of variance (ANOVA one-way).

## 4. Conclusions

In vitro assays concerning the growth of the phytopathogen fungus *F. graminaerum* with CEO added in different concentrations reveals the high antifungal potential of this oil, where the minimum concentration with fungistatic effect is 0.4% and the minimum fungicidal concentration 0.6% CEO. The association of CEO with SEO leads to an enhanced antifungal effect, as shown in the in vivo experiment for *F. graminearum*. Simultaneously, lesser amounts of DON, determined after 14 days, lead to the conclusion that the chemical compounds of EOs exhibit an antagonistic effect on the synthesis of DON-type mycotoxins. Applying the CEO to weed seeds by fumigation reveals the herbicidal potential of this EO, and the effect is amplified by mixing CEO with SEO.

In conclusion, the current study shows the potential of coriander essential oil as an antifungal and herbicidal agent, either as a standalone or in mixtures with SEO. Furthermore, it could be shown that the herbicidal effect and weed seed germination inhibition of CEO is enhanced due to the synergistic effect of the chemical components. Therefore, the CEO used alone or in mixtures with other EOs could be a useful alternative for organic farming.

## Figures and Tables

**Table 1:** The chemical composition of coriander essential oil (CEO) (% of total).

Nr.	Compounds	Type	Retention Time	LRIc *	LRIr **	%
1.	α-Pinene	MH	5.552	1011	1015	11.626
2.	Camphene	MH	6.500	1057	1057	1.083
3.	β-Pinene	MH	7.445	1097	1096	0.710
4.	β-Myrcene	MH	8.684	1164	1164	1.504
5.	d-limonene	MH	9.639	1188	1189	9.628
6.	*o*-Cymene	MH	10.394	1203	1205	0.447
7.	γ-Terpinene	MH	10.757	1238	1238	5.236
8.	*p*-Cymene	MH	11.344	1257	1261	8.000
9.	α-Terpinolene	MH	11.645	1278	1274	0.970
10.	Methyl-heptenone	MO	12.789	1323	1332	0.050
11.	l-Fenchone	MO	14.352	1418	1419	0.237
12.	*trans*-Linalool oxide	MO	15.292	1453	1456	2.817
13.	*cis*-Linalool oxide	MO	15.940	1471	1472	2.284
14.	Camphor	MO	17.086	1507	1511	6.016
15.	Linalool	MO	17.549	1533	1537	45.387
16.	Bornyl-acetate	MO	18.419	1542	1544	0.056
17.	Estragole	MO	20.128	1654	1652	0.158
18.	α-Terpineol	MO	21.116	1694	1692	0.122
19.	Geranyl acetate	MO	21.682	1763	1765	1.423
20.	*p*-Propenil-anisol	MO	23.148	1817	1818	1.630
21.	Thymol	MO	29.948	2160	2164	0.376
Total of major compounds						99.760%
Monoterpene hidrocarbonates (MH)						39.204%
Monoterpene oxygenate (MO)						60.556%

* calculated values of linear retention index, ** literature values of linear retention index [30].

## References

[1] Fleurat-Lessard F. (2017). Integrated management of the risks of stored grain spoilage by seedborne fungi and contamination by storage mould mycotoxins—An update. J. Stored Prod..

[2] Isman M.B. (2000). Plant essential oils for pest and disease management. Crop. Prot..

[3] Flamini G. (2012). Natural Herbicides as a Safer and More Environmentally Friendly Approach to Weed Control: A Review of the Literature Since 2000. Struct. Chem..

[4] Laribi B., Kouki K., M’Hamdi M., Bettaieb T. (2015). Coriander (*Coriandrum sativum* L.) and its bioactive constituents. Fitoterapia.

[5] Singletary K. (2016). Coriander: Overview of Potential Health Benefits. Nutr. Today.

[6] Silva F., Ferreira S., Duarte A., Mendonça D.I., Domingues F.C. (2011). Antifungal activity of Coriandrum sativum essential oil, its mode of action against Candida species and potential synergism with amphotericin B. Phytomedicine.

[7] Freires I.D.A., Murata R.M., Furletti V.F., Sartoratto A., De Alencar S.M., Figueira G.M., Rodrigues J.A.D.O., Duarte M.C.T., Rosalen P.L. (2014). *Coriandrum sativum* L. (Coriander) essential oil: antifungal activity and mode of action on Candida spp., and molecular targets affected in human whole-genome expression. PLoS ONE.

[8] Žabka M., Pavela R., Prokinova E. (2014). Antifungal activity and chemical composition of twenty essential oils against significant indoor and outdoor toxigenic and aeroallergenic fungi. Chemosphere.

[9] Zardini H.Z., Tolueinia B., Momeni Z., Hasani Z., Hasani M., Branch Y. (2012). Analysis of antibacterial and antifungal activity of crude extracts from seeds of Coriandrum sativum. Gomal J. Med. Sci..

[10] Morais W., Lima M., Zanuncio J., Oliveira M., Bragança M., Serrão J., Della Lucia T., Lima M.A. (2015). Extracts of *Ageratum conyzoides*, *Coriandrum sativum* and *Mentha piperita* inhibit the growth of the symbiotic fungus of leaf-cutting ants. Ind. Crop. Prod..

[11] Ferrigo D., Raiola A., Causin R., McPhee D.J. (2016). Fusarium Toxins in Cereals: Occurrence, Legislation, Factors Promoting the Appearance and Their Management. Molecules.

[12] Manganyi M., Regnier T., Olivier E. (2015). Antimicrobial activities of selected essential oils against *Fusarium oxysporum* isolates and their biofilms. S. Afr. J. Bot..

[13] Matusinsky P., Zouhar M., Pavela R., Novy P. (2015). Antifungal effect of five essential oils against important pathogenic fungi of cereals. Ind. Crop. Prod..

[14] Seseni L., Regnier T., Der Merwe M.R.-V., Mogale E., Badenhorst J. (2015). Control of Fusarium spp. causing damping-off of pine seedlings by means of selected essential oils. Ind. Crop. Prod..

[15] Ullah N., Parveen A., Bano R., Zulfiqar I., Maryam M., Jabeen S., Liaqat A., Ahmad S. (2016). In vitro and in vivo protocols of antimicrobial bioassay of medicinal herbal extracts: A review. Asian Pac. J. Trop..

[16] Ferdes M., Al Juhaimi F., Özcan M., Ghafoor K. (2017). Inhibitory effect of some plant essential oils on growth of *Aspergillus niger*, *Aspergillus oryzae*, *Mucor pusillus* and *Fusarium oxysporum*. S. Afr. J. Bot..

[17] Sumalan R.-M., Alexa E., Poiana M.-A. (2013). Assessment of inhibitory potential of essential oils on natural mycoflora and *Fusarium* mycotoxins production in wheat. Chem. Cent. J..

[18] De Almeida L.F.R., Frei F., Mancini E., De Martino L., De Feo V. (2010). Phytotoxic Activities of Mediterranean Essential Oils. Molecules.

[19] Calo J.R., Crandall P.G., O’Bryan C.A., Ricke S.C., O’Bryan C.A. (2015). Essential oils as antimicrobials in food systems – A review. Food Control.

[20] Alexa E., Sumalan R.M., Danciu C., Obistioiu D., Negrea M., Poiana M.-A., Rus C., Radulov I., Pop G., Dehelean C. (2018). Synergistic Antifungal, Allelopatic and Anti-Proliferative Potential of *Salvia officinalis* L., and *Thymus vulgaris* L. Essential Oils. Molecules.

[21] Bakkali F., Averbeck S., Averbeck D., Idaomar M. (2008). Biological effects of essential oils – A review. Food Chem. Toxicol..

[22] Mandal S., Mandal M. (2015). Coriander (*Coriandrum sativum* L.) essential oil: Chemistry and biological activity. Asian Pac. J. Trop. Biomed..

[23] Zamindar N., Sadrarhami M., Doudi M. (2016). Antifungal activity of coriander (*Coriandrum sativum* L.) essential oil in tomato sauce. J. Food Meas. Charact..

[24] Momin A., Acharya S., Gajjar A. (2012). *Coriandrum sativum*. Review of advances in phytophaymacology. Intern. J. Pharm. Sci. Res..

[25] Soares B.V., Morais S.M., Fontenelle R.O.D.S., Queiroz V.A., Vila-Nova N.S., Pereira C.M.C., Brito E.S., Neto M.A.S., Brito E.H.S., Cavalcante C.S.P. (2012). Antifungal Activity, Toxicity and Chemical Composition of the Essential Oil of *Coriandrum sativum* L. Fruits. Molecules.

[26] Al-Snafi P.D.A.E. (2016). A review on chemical constituents and pharmacological activities of *Coriandrum sativum*. IOSR J. Pham..

[27] Darughe F., Barzegar M., Sahari M.A. (2012). Antioxidant and antifungal activity of Coriander (*Coriandrum sativum* L.) Essential oil in cake. Intern. Food Res. J..

[28] Nurzyńska-Wierdak R. (2013). Essential oil composition of the coriander (*Coriandrum sativum* L.) herb depending on the development stage. Acta Agrobot..

[29] Yildiz H. (2016). Chemical composition, antimicrobial, and antioxidant activities of essential oil and ethanol extract of *Coriandrum sativum* L. leaves from Turkey. Intern. J. Food Prop..

[30] Strobl H., Kolodziejczyk P. (2004). 14-Methylpentadecano-15-lactone (Muscolide): A New Macrocyclic Lactone from the Oil of *Angelica archangelica* L.. Chem. Biodivers..

[31] Ateș D.A., Erdogrul O.T. (2003). Antimicrobial Activities of Various Medicinal and Commercial Plant Extracts. Turk. J. Biol..

[32] Zare-shehneh M., Askarfarashah M., Ebrahimi L. (2014). Biological activities of a new antimicrobial peptide from *Coriandrum sativum*. Intern. J. Biosci..

[33] Wang H., Yang Z., Ying G., Yang M., Nian Y., Wei F., Kong W. (2018). Antifungal evaluation of plant essential oils and their major components against toxigenic fungi. Ind. Crop. Prod..

[34] Andrés M., Rossa G., Cassel E., Vargas R., Santana O., Díaz C., Gonzalez-Coloma A. (2017). Biocidal effects of Piper hispidinervum (Piperaceae) essential oil and synergism among its main components. Food Chem. Toxicol..

[35] Goger G., Demirci B., Ilgın S., Demirci F. (2018). Antimicrobial and toxicity profiles evaluation of the Chamomile (*Matricaria recutita* L.) essential oil combination with standard antimicrobial agents. Ind. Crop. Prod..

[36] Boukaew S., Prasertsan P., Sattayasamitsathit S. (2017). Evaluation of antifungal activity of essential oils against aflatoxigenic *Aspergillus flavus* and their allelopathic activity from fumigation to protect maize seeds during storage. Ind. Crop. Prod..

[37] Katar D., Kacar O., Kara N., Aytaç Z., Göksu E., Kara S., Elmastaş M. (2017). Ecological variation of yield and aroma components of summer savory (*Satureja hortensis* L.). J. Appl. Res. Med. Aromat. Plant..

[38] Velluti A. (2003). Inhibitory effect of cinnamon, clove, lemongrass, oregano and palmarose essential oils on growth and fumonisin B1 production by *Fusarium proliferatum* in maize grain. Int. J. Food Microbiol..

[39] Radhakrishnan R., Alqarawi A.A., Abd_Allah E.F. (2018). Bioherbicides: Current knowledge on weed control mechanism. Ecotoxicol. Environ. Saf..

[40] Atak M., Mavi K., Uremis I. (2016). Bio-herbicidal effects of oregano and rosemary essential oils on germination and seedling growth of bread wheat cultivars and weeds. Rom. Biotechnol. Lett..

[41] Aslani F., Juraimi A.S., Ahmad-Hamdani M.S., Hashemi F.S.G., Alam M.A. (2016). Control of weeds in glasshouse and rice field conditions by phytotoxic effects of *Tinospora crispa* (L.) Hook. f. & Thomson leaves. Chil. J. Agric. Res..

[42] Paudel V.R., Gupta V.P. (2009). Effect of some Essential Oils on Seed Germination and Seedling Length of *Parthenium hysterophorous* L.. Ecoprint: Int. J. Ecol..

[43] Campiglia E., Mancinelli R., Cavalieri A., Caporali F. (2007). Use of Essential Oils of Cinnamon, Lavender and Peppermint for Weed Control. Ital. J. Agron..

[44] European Pharmacopoeia Commission (1975). European Pharmacopoeia.

[45] Babushok V.I., Linstrom P.J., Zenkevich I.G. (2011). Retention Indices for Frequently Reported Compounds of Plant Essential Oils. J. Phys. Chem. Ref. Data.

[46] Dwivedi S.K., Sangeeta (2015). Efficacy of some medicinal plant extract against *Fusarium oxysporum* ssp. ciceri causing chickpea wilt. Asian J. Crop Sci..

[47] Alexa E., Danciu C., Cocan I., Negrea M., Morar A., Obistioiu D., Dogaru D., Berbecea A., Radulov I. (2018). Chemical Composition and Antimicrobial Potential of *Satureja hortensis* L. in Fresh Cow Cheese. J. Food Qual..

[48] Doolotkeldieva T. (2010). Microbiological Control of Flour-Manufacture: Dissemination of Mycotoxins Producing Fungi in Cereal Products. Microbiol. Insights.

